# Effects of aging and calorie restriction on the global gene expression profiles of mouse testis and ovary

**DOI:** 10.1186/1741-7007-6-24

**Published:** 2008-06-03

**Authors:** Alexei A Sharov, Geppino Falco, Yulan Piao, Suresh Poosala, Kevin G Becker, Alan B Zonderman, Dan L Longo, David Schlessinger, Minoru SH Ko

**Affiliations:** 1 Laboratory of Genetics, National Institute on Aging, National Institutes of Health, Baltimore, MD 21224, USA; 2 Research Resources Branch, National Institute on Aging, National Institutes of Health, Baltimore, MD 21224, USA; 3 Laboratory of Immunology, National Institute on Aging, National Institutes of Health, Baltimore, MD 21224, USA

## Abstract

**Background:**

The aging of reproductive organs is not only a major social issue, but of special interest in aging research. A long-standing view of 'immortal germ line versus mortal soma' poses an important question of whether the reproductive tissues age in similar ways to the somatic tissues. As a first step to understand this phenomenon, we examine global changes in gene expression patterns by DNA microarrays in ovaries and testes of C57BL/6 mice at 1, 6, 16, and 24 months of age. In addition, we compared a group of mice on *ad libitum *(AL) feeding with a group on lifespan-extending 40% calorie restriction (CR).

**Results:**

We found that gene expression changes occurred in aging gonads, but were generally different from those in somatic organs during aging. For example, only two functional categories of genes previously associated with aging in muscle, kidney, and brain were confirmed in ovary: genes associated with complement activation were upregulated, and genes associated with mitochondrial electron transport were downregulated. The bulk of the changes in gonads were mostly related to gonad-specific functions. Ovaries showed extensive gene expression changes with age, especially in the period when ovulation ceases (from 6 to 16 months), whereas testes showed only limited age-related changes. The same trend was seen for the effects of CR: CR-mediated reversal of age-associated gene expression changes, reported in somatic organs previously, was limited to a small number of genes in gonads. Instead, in both ovary and testis, CR caused small and mostly gonad-specific effects: suppression of ovulation in ovary and activation of testis-specific genes in testis.

**Conclusion:**

Overall, the results are consistent with unique modes of aging and its modification by CR in testis and ovary.

## Background

Aging is a complex biological phenomenon characterized by a gradual and progressive loss of function in diverse organs and tissues. Global gene expression profiling is beginning to provide molecular signatures of aging in various organs and tissues [1,2]. For example, aged muscles show a reduced expression of genes involved in energy metabolism and an increased expression of heat-shock proteins and oxidative-stress inducible genes [3]. The most recent analysis of human muscle samples has revealed the increased expression of genes associated with extracellular matrix, cell growth, complement activation, and cytosolic ribosomes, as well as the decreased expression of genes involved in chloride transport and mitochondrial electron transport [1]. Aged brain tissues show increased expression of genes associated with stress and the inflammatory response and decreased expression of genes associated with the ubiquitin-proteasome pathway and growth factors [2,4]. Aged heart shows increased mRNA levels for structural proteins and decreased mRNA levels for genes involved in fatty-acid metabolism [3]. Global gene expression profiling has also revealed that age-related gene expression changes in muscle are completely [5] or partially [6] suppressed by calorie restriction (CR), which is a factor capable of increasing the lifespan in organisms, including rodents [7-9]. Similarly, it has been shown that age-related decrease in the induction of Hsp70 in response to heat stress in rat hepatocytes is partially reversed by CR [10].

Gonads, especially ovary, are of special interest in aging research, because the human ovary shows an especially demarcated effect of age: a reproducible decline of reproductive capacity in the mid-30 s, followed by menopause in the late 40 s to 50 s. Modern trends postponing childbearing in developed countries are thus accompanied by increased infertility and other complications. Aging of gonads also has direct implications for longevity, as increased fertility is associated with decreased longevity in several species [11,12]. An increase in longevity in mutants is often accompanied by a reduction of fecundity, whereas delayed reproduction is often associated with longevity. For example, it has been shown that the ablation of germ cells increases the lifespan of the worm [13] (although not the fly [14]). Furthermore, the transplantation of young ovary increases the lifespan in mouse, suggesting that ovaries can affect the lifespan of organisms, possibly through the production of hormones and other factors [15]. Gonads are also the focus of attention in various theories of aging [16]. For example, an evolutionary theory of aging considers that aging occurs because the maintenance of individuals is not supported by natural selection after their reproductive periods [11]. The disposable soma (DS) theory suggests that when available energy is limited, it is selectively reallocated from reproduction to somatic maintenance, thermogenesis, DNA repair and anti-oxidative functions [7,11,17].

Despite the importance of gonads in aging research, studies of gonad aging by global gene expression profiling have been limited to partial studies of mouse oocytes [18] and ovary [19]. It has remained unclear whether gonads and somatic organs show similar patterns of gene expression associated with aging and CR. In this study, we have carried out global gene expression profiling of testis and ovary from 1- to 24-month-old mice on *ad libitum *(AL) or CR diets. The whole-genome DNA microarray data provide a resource for the analysis of combined effects of age, diet, and sex on gene expression in the gonads.

## Results

### Experimental design

**Figure 1:**
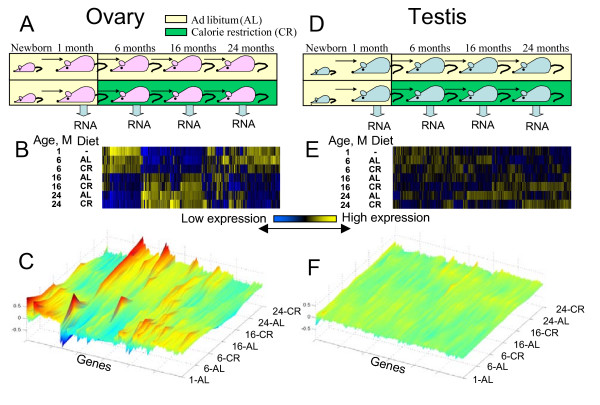
**Global patterns of gene expression in ovary and testis in mice of age from 1 to 24 months, and on an AL or a CR diet**. (A) Experimental design for females; (B) two-dimensional heatmap of expression of 3000 most significant genes in ovary; (C) three-dimensional heatmap of expression of the same genes in ovary; (D) experimental design for males; (E) two-dimensional heatmap of expression of 3000 most significant genes in testis (819 genes overlapped with ovary); (F) three-dimensional heatmap of expression of the same genes in testis.

We analyzed gene expression patterns in ovaries and testes sampled from 1-, 6-, 16-, and 24-month-old C57BL/6 mice fed on AL and CR diets (Figure 1A and 1D). Mice were kept on an AL diet until 14 weeks of age and then split into AL and CR gonads [20]. Two individual animals for each condition were used for the microarray studies. We examined a total of 28 samples (two AL ovaries at each of 1, 6, 16, and 24 months; two CR ovaries at 6, 16, and 24 months; two AL testes at 1, 6, 16, and 24 months; and two CR testes at 6, 16, and 24 months). These 28 samples were hybridized on whole-genome oligonucleotide microarrays bearing approximately 44,000 probes, representing 25,585 nonredundant genes with gene symbols [21] (Additional file 1). The same RNA samples were used for quantitative reverse-transcription polymerase chain reaction (RT-PCR) validation of selected genes.

The effects of CR on physiological status and overall survival curve for C57BL/6 mice used in this study have been well established [20]. For example, it has been shown that 40% CR suppresses follicle cycles and ovulation in female C57BL/6 mice [22]. Survival data on the oldest C57BL/6 mice we used (24 months) are as follows: males AL (70%), females AL (60%), males CR (90%), and females CR (85%) [20]. The average lifespan of this strain on AL diet is around 30 months; the CR diet extends average lifespan up to 50% [20].

### Global analysis of age-related changes in gene expression: major changes in ovary and minor changes in testis

We first analyzed the global gene expression changes with age by analysis of variance (ANOVA; see Methods for the details) and principal component analysis (PCA; Additional file 2). The most significantly altered 3000 genes in ovary and testis were then selected and presented in the two-dimensional and three-dimensional heatmaps. The heatmaps revealed dramatic gene expression changes in ovary (Figure 1B and 1C), whereas testis showed strikingly limited changes in gene expression over time (Figure 1E and 1F). There was a major alteration of gene expression patterns in ovary between 6 and 16 months (Figure 1B and 1C), which corresponds to the time when ovulation ceases in mice [19]. A simple pair-wise comparison between samples from the youngest (1 month) and the oldest (24 months) animals also revealed a difference in the rate of change between testis and ovary. There were 4657 genes with significant differential expression between oldest (24 months) and youngest (1 month) females on AL diet; and 1724 of these genes had more than two-fold change in their expression. In contrast, there were only 326 genes with significant differential expression between the oldest (24 months) and youngest (1 month) males on AL diet; and only 122 of these genes had more than two-fold change in their expression. It was also clear from the heatmaps that the difference between AL and CR in each age group was modest. Thus, CR did not suppress age-associated gene expression changes in testis and ovary.

To examine organ-specific genes, we first separated genes that were predominantly expressed in ovary or testis from genes that were expressed in both organs. There were 14,438 genes with differential expression between ovary and testis (false discovery rate (FDR) at most 0.1, at least 1.5-fold change in gene expression; see Additional file 3). Genes that had a more than two-fold difference in expression between ovary and testis (*N *= 9993), are called 'ovary-associated' and 'testis-associated' genes throughout the analysis below.

### Major trends in gene expression changes with age in ovary

**Figure 2:**
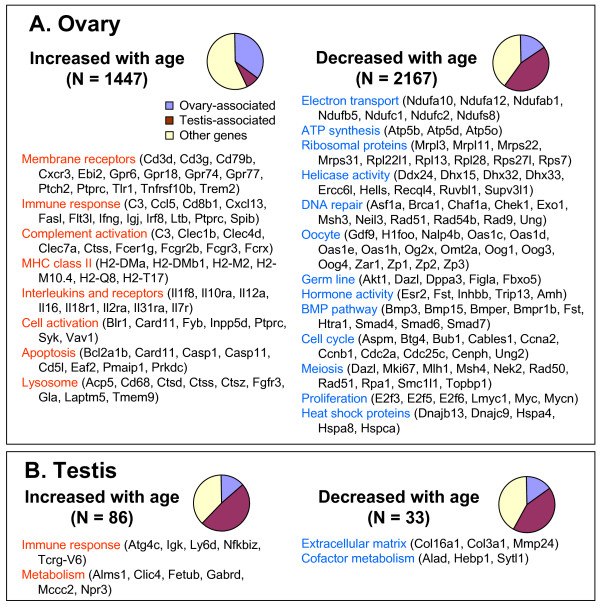
**Functional groups of genes affected by age**. (A) Ovary and (B) testis. Pie charts show the proportion of genes that were over-expressed either in ovary or testis by at least two-fold. Functional categories of genes were assembled from GO annotations and from PubMed.

To focus on the differences between young and old ovary, we compared a group of samples from young (1 and 6 months) females with a group of samples from old (16 and 24 months) females on AL diet. Based on one-factor ANOVA we found 3937 genes that were differentially expressed between young and old ovaries (FDR at most 0.1, at least 1.5-fold change in gene expression level); however, 323 of them did not pass the two-factor linear regression test (*p *< 0.05 for age variable) and therefore were not considered age-dependent. There were 1447 genes that increased and 2167 genes that decreased their expression in the ovary with age, respectively (Figure 2A and Additional file 4). To further characterize these genes, we analyzed functional groups of genes in these sets based on gene ontology (GO) [23] terms (Additional file 5) and information gleaned from the literature. Examples of genes that showed the high differential expressions during aging and were grouped into different functional groups are presented in Additional file 6.

Groups of genes that increased their expression with age included 'receptor binding' (*N *= 43; mostly membrane receptors), 'defense response' (*N *= 83), and 'immune response' (*N *= 65); see Additional file 5. Examples of genes related to immune response included interleukins and interleukin receptors (Additional file 6A), and major histocompatibility complex (MHC) II (Additional file 6B). MHC-II genes are known to be expressed in the theca layer of growing ovarian follicles, and their expression is increased in postovulatory follicles [24]. The expression of apoptosis-related genes (for example, *Bcl2a1b*, *Casp1*, *Casp11*) and several tumor necrosis factor (TNF) receptors (Additional file 6E and 6F) also increased with age. Many genes were related to complement activation (innate immune response), including Fc receptors (*Fcrx*, *Fcgr2b*, *Fcgr3*, *Fcer1g*), complement components (*C3*, *C1sb*), lectins (*Clec7a*, *Clec4d*, *Clec1b*, *Clec4a3*, *Lgals3*; Additional file 6D), toll-like receptors (*Tlr1*, *Tlr2*, *Tlr13*), and cathepsins (*Ctss*, *Ctsd*, *Ctsb*, *Ctsz*).

Groups of genes that decreased their expression with age included 'mitochondrion' (*N *= 101), 'protein biosynthesis' (*N *= 98), 'RNA metabolism' (*N *= 91), 'DNA metabolism' (*N *= 128), 'cell cycle' (*N *= 119), and 'response to DNA damage' (*N *= 47) (Figure 2A, Additional files 5, 6J and 6L). The mitochondrion group included electron transport, adenosine triphosphate (ATP) synthesis, and mitochondrial ribosomal proteins. The protein biosynthesis group included cytosolic and mitochondrial ribosomal proteins. Taken together, a general decline of metabolism is one of the main features of ovary aging.

Groups of genes that decreased their expression with age also included many oocyte-specific genes: *Oog1*, *Oog3*, *Oog4*, *Zp1*, *Zp2*, *Zp3*, *Nalp5 (Mater)*, *Gdf9*, *Og2x*, *Omt2a*, *H1foo*, *Oas1d*, *Zar1*, *Nalp4b*, and *Oosp1 *(Figure 2A and Additional file 6G, H and 6I). Oogenesin3 (*Oog3*) showed the most dramatic decrease in expression (57.7-fold) from age 1 to 24 months. In our earlier comparison of young and old oocytes [18], expression of some of these genes in oocytes decreased with age (for example, *Nalp5*, *Zp3*) but no more than three-fold from 5–6 weeks to 42–45 weeks. The current data indicate a larger decrease of expression of these genes in the ovary (for example, 8-fold for *Nalp5 *and 5.3-fold for *Zp3 *within a comparable age interval from 1 to 16 months), which is most likely related to the reduction of the number of oocytes in ovaries with age [25]. Other groups of genes that decreased expression with age were hormones and hormone receptors, germ-line-specific genes, genes associated with cell cycle, the bone morphogenetic protein (BMP) pathway, helicase activity, meiosis, and heat shock proteins (Figure 2A and Additional file 6J and 6K). Taken together, these gene expression data are consistent with the characteristic decrease in the number of oocytes and follicles in ovaries with age.

### Major trends in gene expression changes with age in testis

We carried out a similar analysis on testis. Comparison of a group of samples from young (1 and 6 months) males with a group of samples from old (16 and 24 months) males on AL diet with one-factor ANOVA revealed 163 differentially expressed genes (FDR at most 0.1, at least 1.5-fold change in gene expression); however 41 of them did not pass the two-factor linear regression test (*p *< 0.05 for age variable) and therefore were not considered age-dependent. There were 86 genes that increased and 33 genes that decreased their expression in testis with age (Figure 2B and Additional file 7). Only 10 up-regulated genes and 9 down-regulated genes changed their expression consistently in testis and ovary (Additional file 7), which indicates that major age-related changes of gene expression in testis are mostly organ-specific. Indeed, age-affected genes in testis included a large portion of testis-associated genes (see the pie charts in Figure 2B). Genes that increased their expression with age included immune response and metabolism (Figure 2B and Additional file 8A and 8B). Genes that decreased their expression in testis with age included extracellular matrix and cofactor metabolism (Figure 2B; Additional files 8C and 9).

### Effect of CR on gene expression in ovary

**Figure 3:**
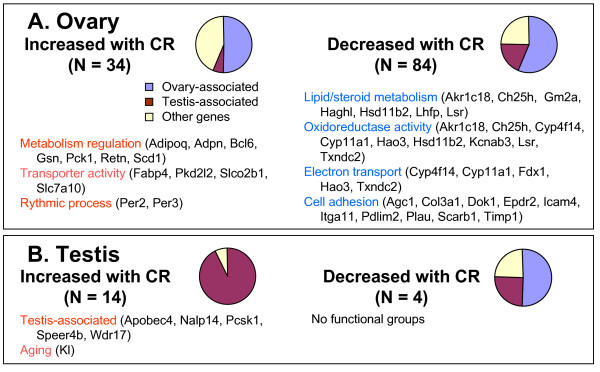
**Functional groups of genes affected by diet (AL versus CR)**. (A) Ovary and (B) testis. Pie charts show the proportion of genes that were over-expressed either in ovary or testis by at least two-fold. Functional categories of genes were assembled from GO annotations and from PubMed.

To focus on the differences between AL and CR in ovary, we compared a group of ovary samples from females on a CR diet with a group of ovary samples from females on AL diet using one-factor ANOVA as described in Methods and found 123 differentially expressed genes (FDR at most 0.1, at least 1.5-fold change). However, five of these genes did not pass the additional two-factor ANOVA test (*p *< 0.05 for diet factor) and therefore were not considered diet-dependent. This analysis confirmed our observation that CR affected a much smaller set of genes than aging in ovary (Figure 1B), and identified only 34 genes with increased expression and 84 genes with decreased expression by CR (Figure 3A and Additional file 10).

Consistent with the suppression of ovulation by CR [22], genes that were suppressed by CR in the ovary included *Sfrp4*, *Sgk*, *Lhcgr*, and *Cyp11a1*, which are related to ovarian follicle growth and degradation [26-28]. We also noticed that many genes expressed specifically in oocytes showed slower decline in their expression in mice on CR diet than in mice on AL diet (for example, *Oog1*, *Oog3*, *Oog4*, *Zp2*, *Zp3*, *Nalp5*, *Gdf9*, *Og2x*; see Additional file 6H, I and 6J). Although the difference was not significant based on our statistical criteria (FDR at least 0.1, at most 1.5-fold change), the average log-ratio (CR/AL) of the expression of 25 known oocyte-specific genes [29-32] was significantly positive (0.061 ± 0.015, *p *= 0.0006; see Additional file 11). These data are consistent with the retention of a larger number of oocytes in ovaries of CR mice compared with ovaries of AL mice, as implicated by the suppression of ovulation under CR [22]. However, alternative explanations, such as structural changes other than oocyte numbers in CR ovaries, cannot be excluded.

The expression of metabolism-related genes in the ovary decreased under CR. For example, many mitochondrial genes, including those involved in electron transport, oxidoreductase activity, as well as lipid and sterol-metabolism, decreased their expression in the ovaries of mice on a CR diet (Figure 3A and Additional files 12, 13D, and 13F). Cell adhesion gene expression was also suppressed by CR diet (Figure 3A and Additional files 12 and 13E). On the other hand, CR diet increased the expression of metabolism-related genes (for example, *Adipoq*, *Adpn*, *Fabp4*, *Gsn*, *Mc2r*, *Pck1*, *Per2*, *Per3*, *Retn*, *Scd1*; see Figure 3A and Additional files 12, 13B, and 13C), many of which are known to regulate body weight and food intake. For example, *Retn *and *Scd1 *cause insulin resistance [33,34] and *Scd1 *is induced by a fat-free diet [35]. Adiponectin (*Adipoq*) promotes fatty acid oxidation and glucose uptake and has anti-inflammatory and antiatherogenic effects [36,37]. Gelsolin (*Gsn*) has anti-apoptotic function [38]. *Mc2r *regulates steroidogenesis [39]. *Pck1 *stimulates hepatic glucose output [40]. *Fabp4 *is a transporter of fat molecules [41]. Activation of circadian clock genes (*Per2*, *Per3*) may be related to the regular timing of food supply in CR mice. It has been shown that the *Per2 *gene is one of the major components of the ovarian circadian clock in rats [42].

As an alternative approach to analyzing the data, we compared gene expression levels in ovary between AL and CR conditions for each age group separately, because the above-mentioned analysis comparing all-age pooled data might miss genes responsive to CR in an age-dependent manner. As this analysis was based on two replications only, we expected to see a higher number of false positives. To limit their numbers, we excluded genes that showed contradictory responses to CR at different ages (for details see Methods). By using pair-wise comparisons of log-expression mean values for each age group based on the error variance identified in ANOVA, we found that some metabolism-related genes (*Acacb*, *Dgat2*, *Ces1*, *Ces3*, *Pfkfb3*, and *Pdha1*) were upregulated by CR only in old age (24 months; see Additional file 14). It has been reported that *Dgat2 *is regulated by Leptin and is involved in insulin resistance [43]. *Acacb *and *Pfkfb3 *are also involved in the regulation of metabolism [44-46]. In contrast, the expression of relaxin receptor *Lgr7*, which stimulates appetite in rats [47] and dystrophin related protein *Drp2 *was upregulated by CR only in young females. Similarly, a large group of genes (*N *= 166) were downregulated by CR only in the ovary of young females (6 months). These genes included many metabolism genes (for example, *Akr1c18*, *Acox3*, *Ch25h*, *Cox6a1*, *Fdx1*, *Olr1*, *Pigt*, *Sdc1*) and genes that are expressed in ovarian follicles (*Cdkn1b*, *Cdkn2b*, *Mif*, *S100a6*, *Saa1*, and *Star*) [27,48-51]. This group of genes also included *Sfrp4*, which is expressed in corpus luteum [52], genes involved in extracellular matrix (*Adamts4*, *Adamts12*, *Col3a1*, *Col4a1*, *Lamc1*, *Mmp2*, *Mmp9*, *Sparc*, *Timp1*), and nucleosome assembly (*Hist1h2aa*, *Hist1h2bn*, *Hist1h4d*, *Hist1h4h*, *Hist1h4m*, *Hist2h4*, *Myst3*), which may be indirectly related to follicle growth and degradation. The decrease of expression of these genes is consistent with the repression of ovulation by CR in young females.

**Figure 4:**
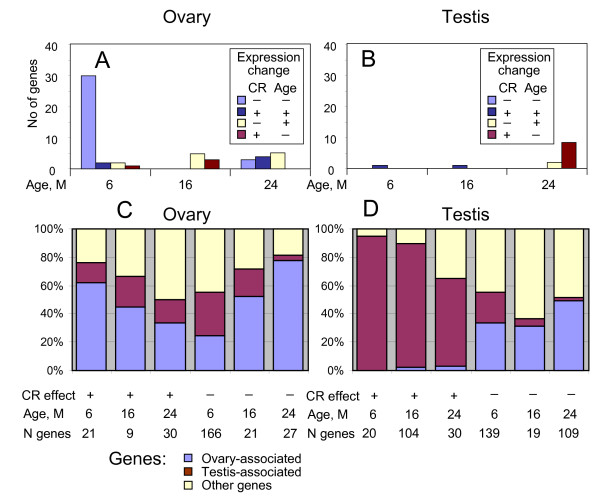
**Age-specific effect of diet (AL versus CR)**. (A), (B) Numbers of age-dependent genes which were also affected by diet at specific age from 6 to 24 months in ovary and testis, respectively. (C), (D) Proportion of genes that were over-expressed either in ovary or testis, respectively, by at least two-fold among genes that were increased or decreased by CR at specific age from 6 to 24 months.

As mentioned in the introduction, it is of major interest whether CR can delay or abolish age-associated alterations of gene expression. However, the number of genes altered in the ovary both by age and CR over the entire lifespan was very small (*N *= 18; see Additional file 10), which suggests a lack of interaction between these processes. However, we found more interaction between CR and aging by considering age-specific effects of CR in ovaries (Figure 4 and Additional file 14). Among 35 genes affected by both age and CR at 6 months, the majority (91%, *N *= 32) changed their expression in the same direction in old age and under CR (Figure 4A). As most of these genes decreased with both age and CR (*N *= 30) and included genes related to follicle growth and ovulation (see above), we suggest that CR at a young age causes premature decline of ovarian function, especially repression of follicle growth and ovulation. However, at older ages CR caused some anti-aging effects in ovaries: of 20 genes whose expression depended on both age and diet at the age of 16 or 24 months, 13 genes changed their expression in the opposite direction in response to age and CR (Figure 4 and Additional file 14). For example, *Clec4d*, *Crb2*, *Csprs*, *Lefty1*, *Mcpt5*, *Sfrp4*, and *Upk1b *increased their expression with age but were suppressed by CR.

### Effect of CR on gene expression in testis

We next focused on the differences between AL and CR in testis, and compared a group of samples from males on a CR diet with a group of samples from males on an AL diet using one-factor ANOVA (as described in Methods) and found 21 differentially expressed genes (FDR at most 0.1, at least 1.5-fold change). However, two of these genes did not pass the additional two-factor ANOVA test (*p *< 0.05 for diet factor) and therefore were not considered diet-dependent. There were 15 genes that increased and 4 genes that decreased their expression in the testes of mice on a CR diet (Figure 3B and Additional files 13G, H, I, and 15). There was no overlap between genes that responded to CR in ovary and testis, which implies that the effect of CR is specific to testis or ovary, and suggests that there are no universal effects of CR in gonads.

Among genes activated by CR in testis, the majority were testis-associated (93%, *N *= 14; Figure 3B and Additional file 14). These genes included two metabolism-related genes, *Pcsk1*, which is directly involved in converting proinsulin to insulin [53], and *Apobec4*, as well as genes involved in spermatogenesis (*Nalp14*, *Speer4b*) [54,55].

Analysis of age-specific responses of gene expression to CR in testis revealed additional genes affected by diet (Additional file 16). To limit false positives, we excluded genes that showed contradictory responses to CR at different ages (for details see Methods). Genes that were suppressed by CR in testis at various ages included genes associated with the immune response (*Ccl11*, *Ccl8*, *Clec4e*, *Clec7a*, *Cxcl5*, *Gbp4*, *Ifit2*, *Osm*, *Tlr2*, *Tlr7*) and cellular morphogenesis (*Creg1*, *Esm1*, *Krt1-17*, *Nov*, *Osm*). The majority of genes activated by CR were testis-associated, especially at younger ages (6–16 months), whereas only a few testis-associated genes were suppressed by CR (Figure 4D). Over-represented functional groups of genes activated by CR included ion transport (*Catsper4*, *Chrna9*, *Emid2*, *Grin2b*, *Kcnn2*, *Kcnn3*, *Kcnu1*, *Sytl1*) and carboxylic acid metabolism (*Acot12*, *Cpt1b*, *Folh1*, *Gls2*, *Pdgfd*, *Si*, *Tdo2*). These data clearly show that testicular functions are not harmed even under low-energy conditions. There were only 12 genes affected by both age and CR in testis, and the majority of them (*N *= 10) changed in opposite directions with age and CR in the oldest group of males (24 months); see Figure 4B.

### Expression of genes associated with aging, DNA repair, and heat shock response

To look at the data from a different perspective, we examined genes that are known to be associated with aging (for example, *Sirt *gene family [56]) as well as genes involved in repair and control of DNA and the response to heat shock, because they are potential indicators of the aging process. The list of these genes (Additional file 17, *N *= 218) was obtained by combining information from GO annotations, Bioscience Corporation [57] and the literature [58]. Examples of gene expression patterns for these genes are given in Additional file 18. A large portion of these genes contained testis-associated genes (47%, *N *= 103), and only a small portion of genes (10%, *N *= 22) was associated with ovary (Additional file 17). Expression of these genes is probably linked to reproducing germ cells, which are abundant in testis and absent in ovary. For example, Fanconi anemia gene *Fancd2 *and breast cancer gene *Brca2 *had a higher expression in testis than in ovary (Additional file 18A). However, some genes were over-expressed in ovary, including the alpha thalassemia gene *Atrx*, which is known to be involved in chromatin regulation [59] (Additional file 18A). *Sirt1 *and *Sirt2 *had a higher expression in testis than in ovary, and *Sirt6 *was expressed more in ovary than in testis (Additional file 18B). Different sets of telomere-maintenance genes were over-expressed in ovary and testis (Additional file 18C). Testis had over-expression of *Terf1 *and *Terf2*, whereas ovary had a slightly higher expression of *Tep1*.

The majority of genes that were affected by aging in ovary decreased their expression with age (*N *= 67; for example, *Atr*, *Brca1*, *Brca2*, *Sirt6*, and *Fancd2*) and only a few genes (*N *= 8; for example, *Prkdc*, *Dnajc6*, and *Dnajb12*) increased their expression with age (Additional file 17). This result was expected because most DNA repair genes are associated with germ-line cells, which decreased in numbers in ovaries with age. Only a few genes related to aging, DNA repair, and heat shock changed their expression with age in testis, or with diet in either ovary or testis.

## Discussion

Global expression profiling of nearly all genes has captured genome-wide trends in gene expression during aging and its modification by CR in ovary and testis. The current study has revealed large age-related changes of gene expression in ovary compared with very limited changes in testis. In ovary, oocyte- and germ-line-specific genes decreased their expression with age, whereas genes related to membrane receptors and immune response increased their expression with age. The difference between ovary and testis reflects in part the dramatic changes of tissue composition in ovaries than in testes. During the course of life, ovaries undergo progressive changes of tissue compositions owing to changes in the number of follicles and atretic follicles, and in the amount of interstitial and stromal scar tissues, and vasculatures. It is thus important to point out that the gene expression profiles of a whole ovary reflect compounding effects of changes in tissue compositions and changes in gene expression of cells constituting each tissue type.

The current work presents the first global gene expression profile of aging testis, along with a comprehensive profile of aging ovary. Previous reports on aging testis in rodents are limited to a small number of genes (see, for example, [60,61]). Therefore, global gene expression profiles of aging testis in AL and CR conditions will provide useful information for further data mining. In the case of ovary, there have been some reports on global expression profiling of aging ovary and oocytes. Age-associated gene expression changes detected in the current work are comparable with those reported in previous reports. Of 545 genes with symbols that decreased their expression in adult ovaries (4–6 months) compared with newborn ovaries [62], 73 showed decreasing expression with age in our experiments, and only 10 genes showed increasing expression. Some discrepancies in these data sets were expected because different microarray platforms and ages of mice were used in different studies. For example, among the genes identified as differentially expressed in ovaries between 1.5- and 8-month-old mice [19], only a few genes (e.g., *Gata3*) were confirmed in our study.

### Does gonad age in a similar manner to other somatic organs?

A long-standing view of 'immortal germ line versus mortal soma' poses an important question of whether the reproductive tissues age in a similar manner to the somatic tissues. Among the six functional categories of genes commonly altered in human muscle, kidney, and brain [1], our analysis revealed that only two categories consistently changed in ovary. Genes related to complement activation were upregulated and genes related to mitochondrial electron transport chain were downregulated. The similarity in response of genes related to complement activation may be superficial, because many of these genes have ovarian-specific functions related to follicle development, ovulation, and corpus luteum formation and degradation [63,64] rather than defense against pathogens. As for other functional categories, genes associated with extracellular matrix and cytosolic ribosomes decreased their expression with age in ovary (Additional file 5), which is opposite to the trend in somatic organs, whereas the remaining two categories, cell growth and chloride transport, showed no consistent age-related trend in ovaries. In testis, we observed no significant changes in any of these functional groups of genes. Apparently the number of genes affected by age was limited. Taken together, these data indicate that aging of gonads generally shows a different pattern of gene expression changes than aging of somatic organs. Testis ages little by our criteria and thus seems to support the idea of immortal germ line versus mortal soma. Ovary shows dramatic age-associated changes in gene expression with some similarity to somatic organs, but the interpretation of the data is complicated by the fact that ovary aging is dominated by the progressive loss of follicles during the reproductive lifespan. Indeed, our earlier microarray study comparing oocytes isolated from young mice with oocytes isolated from old mice shows only moderate gene expression changes with age: of around 22,000 genes examined there were only 99 genes with a more than two-fold change between old and young oocytes [18].

### Does CR delay age-associated gene expression changes in gonad?

It has been reported that CR can slow down age-associated changes of gene expression. For example, the analysis of muscle showed that age-related changes in expression are mostly suppressed by CR [5]. On the other hand, there has also been a report that such CR-effects on gene expression levels are rather limited [6]. It is thus of great interest to determine whether CR can delay the age-associated changes in our gonad aging data. From a global perspective, the current microarray data indicate that such CR-mediated reversal of age-associated gene expression changes is limited to a small number of genes in ovary and testis. However, the interpretation of the data is complicated, especially for ovary, owing to the fact that CR represses ovulation [22]. Considering the significant effects of long-term arrest of cycles on ovary, it is most likely that the observed gene expression changes are the compounding results of direct CR effects on ovary and indirect ovulation suppression effects.

### Is the effect of CR on gene expression in gonads similar to that in somatic organs?

Comparison of our results with published data on the effect of CR on gene expression in liver [65] and heart [66] showed that sets of genes affected by CR are mostly organ-specific. There were only a few common genes affected in a similar manner by CR in ovary and other organs. Three genes, *Scd1*, *Pck1*, and *Amy1 *(at 6 months), were activated by CR in both ovary and liver [65] and four genes, *Scd1*, *Gsn*, *Sfpq*, and *Inmt*, were activated by CR in both ovary and heart [66]. As *Scd1 *was upregulated by CR in three organs (ovary, liver, and heart) and is known to be activated by a fat-free diet [35], this gene could be a generic marker for CR. However, the major effects of CR in gonads were gonad-specific: suppression of ovulation in ovary and activation of testis-associated genes in testis. For example, *Per2 *and *Per3 *(circadian rhythm genes in ovary) were upregulated by CR (Additional file 13A), and are known to be positively associated with lifespan [67]. Although *Per2 *and *Per3 *seem to be ovarian markers for CR, it is also possible that the regular feeding schedule employed to implement CR, activated genes associated with circadian rhythm in ovary [68].

### Regulated expression of adipokines in ovary

One interesting finding in the current work is the regulated expression of adipokines in ovary. Among four well-known adipokines, Leptin, Adiponectin (*Adipoq*), Resistin (*Retn*), and *Visfatin*, we found that *Adipoq *and *Retn *were expressed in ovary and highly induced by CR. Adipokines, secreted from adipocytes, have been implicated in female fertility and obesity [69]. Similarities between adipocyte and ovarian follicle cells have been suggested by the expression of adipocyte-specific type of fatty acid binding protein 4 (*Fabp4*) in granulosa cells undergoing apoptosis [70]. Expression of leptin in granulosa cells and theca cells has also been shown [71,72]. However, we found for the first time that *Adipoq *and *Retn *are expressed in the ovary. The possibility of detecting transcripts encoding adipokines from contaminated adipocytes can be excluded, because the ovary dissection carefully removed neighboring adipose tissues, which was confirmed later by histological examination.

### Implications for theories of aging

The effect of CR on various organs is often discussed in relation to the theory of optimal energy allocation. According to the DS theory, species with low extrinsic mortality (mammals) tend to invest more energy in soma maintenance under CR conditions (which may result in an increased lifespan), at the cost of low energy allocated to reproduction [7,11,17]. Our data on gene expression seem to support this theory for mouse ovary in two ways: first, by demonstrating that patterns of gene expression changes are mostly ovary-specific and are different from those in somatic organs, which indicates differences in resource allocation strategy between reproduction and somatic functions during animal life; and second, by showing a decrease of metabolism and follicle growth in young CR females. Owing to the competition of reproductive and somatic organs for available energy, we expected that CR would affect different sets of genes, and indeed found that a set of genes affected by CR in ovary had a very limited overlap with a set of genes affected by CR in liver and heart.

In contrast, testis showed expression patterns different from ovary: CR did not suppress mitochondrial function, although some metabolism-related genes were suppressed. The majority of genes upregulated by CR were testis-associated, suggesting that there is no tendency to sacrifice testicular functions under a CR diet. Thus, the strategy of energy allocation between somatic and reproduction functions is different between sexes, and males tend to invest more energy in reproduction than into soma maintenance in CR conditions. Our data are consistent with the physiological observation that male fertility is only slightly reduced in rodents under CR [73]. Thus, evolutionary implementation of DS theory may be especially dimorphic. This poses an interesting question. Is an additional explanation required to account for the difference between males and females in terms of evolutionary basis of aging? For example, it is conceivable that under CR conditions a need to shut down reproductive function is much greater in females than in males, because the lives of both mother and fetus are at a risk in pregnant females. Some theories of aging may need to be reexamined in the light of new data reported in this paper.

## Methods

### Animals

The study was carried out as a part of the AGEMAP (Atlas of Gene Expression in Mouse Aging Project) at the Intramural Research Program of the National Institute on Aging (NIA/NIH) [74-76]. C57BL/6 mice were purchased from the National Institute on Aging (NIA) Rodent Colony at ages of 3 weeks, 5, 15, and 23 months. The mice were kept under the standard AL and CR conditions [20]. In the CR cohorts, the caloric intake was reduced to the 60% level in a stepwise manner over 3 weeks, beginning at 14 weeks of age. After transferring to the mouse facility of the NIA Intramural Research Program, the mice were kept under the same conditions. Mice were individually housed. The mice were euthanized at 1, 6, 16, and 24 months of age, and organs were quickly removed and stored in RNAlater at -20°C. The CR mice were fed between 9 AM and 11 AM everyday, but were not fed on the final day and euthanized before noon. The AD mice were continuously supplied with an excess of food. The use of mice in this project was approved by the NIA-IRP Animal Care and Use Committee.

### Microarray experiments

RNA extraction, labeling, and hybridization on a microarray were performed independently for each mouse with two replications for each combination of age, sex, and diet (14 microarrays were used for ovaries and 14 for testis). Two replications were sufficient for this study because the goal was to depict major trends in the change of gene expression with age and diet rather than to assess the individual variability of gene expression for each gene. The latter task would require many more replications but it was beyond the scope of this work. Genes with high individual variability of their expression appeared not significant in our statistical analysis and therefore were ignored. Most of the analysis (except the age-specific effect of CR) is based on at least four samples which increased the reliability of results. Total RNAs were extracted from entire organ (ovary or testis). The tissue was processed by the Bead Beater (Bio-Spec, Bartlesville, OK) followed by RNA purification using the RNeasy Mini Kit (Qiagen, Valencia, CA; Invitrogen). Total RNAs were labeled with Cy3-CTP. Fluorescently labeled microarray targets were prepared from 2.5 μg aliquots of total RNA samples using a Low RNA Input Fluorescent Linear Amplification Kit (Agilent). A reference target (Cy5-CTP-labeled) was produced from Stratagene Universal Mouse Reference RNA. Targets were purified using an RNeasy Mini Kit (Qiagen), and then quantified on a NanoDrop scanning spectrophotometer (NanoDrop Technologies). cRNA was hybridized to the NIA Mouse 44K microarrays v2.1 and v2.2 (whole genome 60-mer oligo arrays manufactured by the Agilent Technology: designs 012799 and 014117, respectively) [77]. All hybridizations compared one Cy3-CTP-labeled experimental target with the single Cy5-CTP-labeled universal mouse reference (Stratagene) target which was used for normalization. Microarrays were hybridized and washed according to the Agilent protocol G4140-90030. Slides were scanned on an Agilent DNA Microarray Scanner, using standard settings, including automatic PMT adjustment. The microarray data discussed in this publication have been deposited in NCBI Gene Expression Omnibus (GEO) [78] and are accessible through GEO Series accession number GSE7502. The data are also available at the NIA Array Analysis software [79,80].

### Statistical analysis

Statistical analysis of microarray results was done using NIA Array Analysis software [79,80] on the basis of 25,585 non-redundant genes with symbols (Additional file 1). Median center global adjustment was used to remove differences between two batches of replications that were performed with different array versions for the ovary (testis samples were all performed with one version of arrays v2.2). For each gene, we estimated the difference between log-transformed gene expression values obtained with different array versions for samples from mice of the same age and diet, then used the median of these differences as an adjustment to all log-transformed expression values for this gene from arrays of v2.1. If a gene was represented by multiple oligos we selected the oligo with the most significant intensity change. One-factor ANOVA was performed with adjustment of error variances for individual genes so that it did not fall below the moving average error variance for genes with similar intensity, and with FDR method for assessing statistical significance [81]. FDR is already corrected for the number of tested hypotheses. Gene expression difference was considered significant based on the threshold of FDR at most 0.1 and fold change at least 1.5. Effects of age were studied by comparison of gonads in young (1 and 6 months) versus old (16 and 24 months) animals. In addition, we used two-factor linear regression with age and diet as independent variables with a threshold of *p *= 0.05 applied to the effect of age. Genes were considered age-dependent if they passed both tests. Effects of diet were studied by comparison of gonad samples from animals on CR diet with a group of samples from animals on AL diet. In this analysis we combined data from mice of age from 6 to 24 months which were adjusted for age effects as follows: *x*_*adi*_' = *x*_*adi *_- *M*_*a *_+ *M*, where *x*_*adi*_' is the adjusted log-transformed gene expression for age *a*, diet *d*, and replication *i*, *x*_*adi *_is the original log-transformed gene expression for age *a*, diet *d*, and replication *i*, *M*_*a *_is the average log-transformed gene expression for age *a *(diets and replications combined), and *M *is the average log-transformed gene expression for all ages. In addition, we used standard two-factor ANOVA without interaction effects and with a threshold of *p *= 0.05 applied to the effect of diet. Genes were considered diet-dependent if they passed both tests. Age-specific effects of CR were detected by pair-wise comparison of means using the error variance from ANOVA for all ages and diets. To reduce false positives, we excluded genes with age-specific effects of CR if these effects were reversed in another age group. Specifically, a gene was excluded if *x · x*_1 _< 0 and |*x*_1_| > 0.3333 · |*x*|, where *x *is the log ratio of gene expression CR/AL in the age group where diet had the strongest effect, and *x*_1 _is the log ratio of gene expression CR/AL in any other age group. PCA was used to identify major patterns of gene expression [79]. Analysis of GO annotation terms in a selected list of genes was done using the NIA Mouse Gene Index (ver. mm7) software [82] using FDR at most 0.1 and enrichment ratio at least 1.5 as thresholds. Only nonredundant genes with gene symbols were used for analysis. Statistical significance was assessed using the hypergeometric distribution and FDR method, which was adjusted to account for redundant GO categories as described [83].

### RT-PCR analysis

The same microarray hybridization RNA samples together with one additional biological replicate (total, *N *= 3) were used for quantitative reverse-transcription (RT)-PCR. The total RNA was DNAse treated (DNA-free, Ambion, Austin, TX, USA), annealed with random hexamer and reverse transcribed into cDNA with ThermoScript reverse transcriptase (Invitrogen, Carlsbad, CA, USA). PCR primer pairs were designed using Vector NTI software (Invitrogen) and were tested on ovary or testis cDNA with SYBR Green PCR Master Mix (Applied Biosystems, Foster City, CA, USA). Each primer pair was run using a matrix of forward and reverse primers concentrations, and threshold cycle measurements were compared with dissociation curves to determine optimal primer concentrations with high amplicon specificity. Genes were analyzed by quantitative RT-PCR on an ABI 7700 Sequence Detection System (Applied Biosystems). Reactions were set up in a 25 μl volume containing 12.5 μl of SYBR Green PCR Master Mix (Applied Biosystems), 2.0 μl of primers, 0.5 μl of H2O and 10.0 μl of cDNA 5.0 ng/μl. The list of primers and relative concentrations are summarized in Additional file 19 and results are shown in Additional file 20. Thermal cycling was initiated with 2 min incubation at 50°C, followed by a two-step PCR amplification at 95°C for 15 s and 60°C for 40 s repeated 40 times. The amount of target mRNA was determined from the appropriate standard curve and divided by the amount of H2A.2 mRNA for normalization.

## Authors' contributions

AAS carried out the data analysis, prepared figures and tables, and wrote the manuscript. GF carried out RNA extraction and qRT-PCR, and contributed to writing the manuscript. YP carried out RNA labeling and microarray hybridization. SP supervised the maintenance of mouse colony, designed the study, and carried out and analyzed the histology. KGB designed the study and supervised the RNA collection. ABZ designed and supervised the study. DLL conceived, designed, and supervised the study, and reviewed the manuscript. DS supervised the study and edited the manuscript. MSHK supervised the study, contributed to the data analysis, and wrote and finalized the manuscript. All authors participated in the discussion and approved the final manuscript.

## Supplementary Material

Additional file 1 Table of normalized expression of nonredundant genes with symbols in ovary and testis. Click here for file

Additional file 2 **Principal Component Analysis (PCA) of expression of all genes in ovary (A) and testis (B) of mice from 1 to 24 months old on *ad libitum *(AL) or calorie restriction (CR) diet**. In ovary, PC1 is associated with age; PC2 is related to an initial increase of gene expression followed by a decrease in the oldest age; PC3 represents the effect of calorie restriction, which is strongest at the age of 6 months. In testis, PC1 is related to age; PC2 is associated with CR but mostly in the oldest animals. The first two principal components accounted for 58% of variance in testis compared to 74% in ovary, which indicates that PCA patterns for testis were noisier than those for ovary due to very limited changes in gene expression values. Click here for file

Additional file 3 **Table of genes that were differentially expressed between ovary and testis**. Genes with at least two-fold over-expression in ovary (or testis) are called ovary – (or testis) associated genes. Click here for file

Additional file 4 Table of genes with differential expression in ovary between young (1–6 months) and old (16–24 months) females on an AL diet. Click here for file

Additional file 5 Table of GO annotations of genes with differential expression in ovary between young (1–6 months) and old (16–24 months) females on and AL diet. Click here for file

Additional file 6 **Gene expression changes in mouse ovarywith age from 1 to 24 months on *ad libitum *(AL) or calorierestriction (CR) diet**. Panels A-F show genes whose expression increases with age; panels G-L show genes whose expression decreases with age. Genes were selected arbitrarily to represent each functional category, but most of them were genes with the greatest differential expression in each functional category. (A) interleukin-related genes, (B) MHC-II genes, (C) complement component genes, (D) C-lectin genes, (E) apoptosis-related genes, (F) TNF receptor genes, (G) oogenesin genes, (H) zona pellucida genes, (I) other oocytes-specific genes, (J) cell cycle-related genes, (K) BMP-signaling genes, and (L) DNA repair genes. Click here for file

Additional file 7 Table of genes with differential expression in testis between young (1–6 months) and old (16–24 months) males on an AL diet. Click here for file

Additional file 8 **Gene expression changes in mouse testis with age from 1 to 24 months on *ad libitum *(AL) and calorie restriction (CR) diet**. Panels A and B show genes whose expression increases with age; panels C and D show genes whose expression decreases with age. Genes were selected arbitrarily to represent each functional category, but most of them were genes with the greatest differential expression in each functional category. (A) immune response genes; (B) metabolism genes; and (C) extracellular matrix genes. Click here for file

Additional file 9 Table of GO annotations of genes with differential expression in testis between young (1–6 months) and old (16–24 months) males on an AL diet. Click here for file

Additional file 10 Table of genes affected by calorie CR in ovary (ages 6–24 months combined, age effects subtracted). Click here for file

Additional file 11 Table of the effect of CR on the expression of oocyte-specific genes in ovary (ages 6–24 months combined). Click here for file

Additional file 12 Table of GO annotations of genes affected by CR in ovary (ages 6–24 months combined). Click here for file

Additional file 13 **Gene expression changes in mouse ovary (A-F) and testis (G-I) with *ad libitum *(AL) or calorie restriction (CR) diet**. Genes were selected arbitrarily to represent each functional category, but most of them were genes with the greatest differential expression in each functional category. (A) circadian rhythm genes, (B) metabolism regulating genes, (C) transporter activity genes, (D) lipid/steroid metabolism-related genes, (E) cell adhesion genes, (F) electron transport genes, (G) testis-associated genes, (H) aging-related gene, and (I) interferon responsive gene. Click here for file

Additional file 14 Table of genes affected by CR in ovary at specific age. Click here for file

Additional file 15 Table of genes affected by CR in testis (ages 6–24 months combined, age effects subtracted). Click here for file

Additional file 16 Table of genes affected by CR in testis at specific age. Click here for file

Additional file 17 Table of the expression of genes associated with aging, DNA repair, and heat shock response in ovary and testis. Click here for file

Additional file 18 **Expression of genes associated with aging and DNA damage control in ovary and testis in mice from 1 to 24 months old on *ad libitum *(AL) or calorie restriction (CR) diet**. Genes were selected arbitrarily to represent each category. (A) genes involved in human diseases, (B) sirtuin family genes, and (C) telomerase-related genes. Click here for file

Additional file 19 Table of primers for q-PCR. Click here for file

Additional file 20 Quantitative RT-PCR analysis of selected genes in ovary and testis in mice from 1 month to 24 months on AL or CR diet. Click here for file
