# Supplementary material for: Effects of aging and calorie restriction on the global gene expression profiles of mouse testis and ovary
Source: BMC Biol. 2008 Jun 3;6:24. doi: 10.1186/1741-7007-6-24 (PMC2426674; doi:10.1186/1741-7007-6-24)
Supplement: Additional file 2 — Principal Component Analysis (PCA) of expression of all genes in ovary (A) and testis (B) of mice from 1 to 24 months old on ad libitum (AL) or calorie restriction (CR) diet. In ovary, PC1 is associated with age; PC2 is related to an initial increase of gene expression followed by a decrease in the oldest age; PC3 represents the effect of calorie restriction, which is strongest at the age of 6 months. In testis, PC1 is related to age; PC2 is associated with CR but mostly in the oldest animals. The first two principal components accounted for 58% of variance in testis compared to 74% in ovary, which indicates that PCA patterns for testis were noisier than those for ovary due to very limited changes in gene expression values. [file 1741-7007-6-24-S2.pdf]

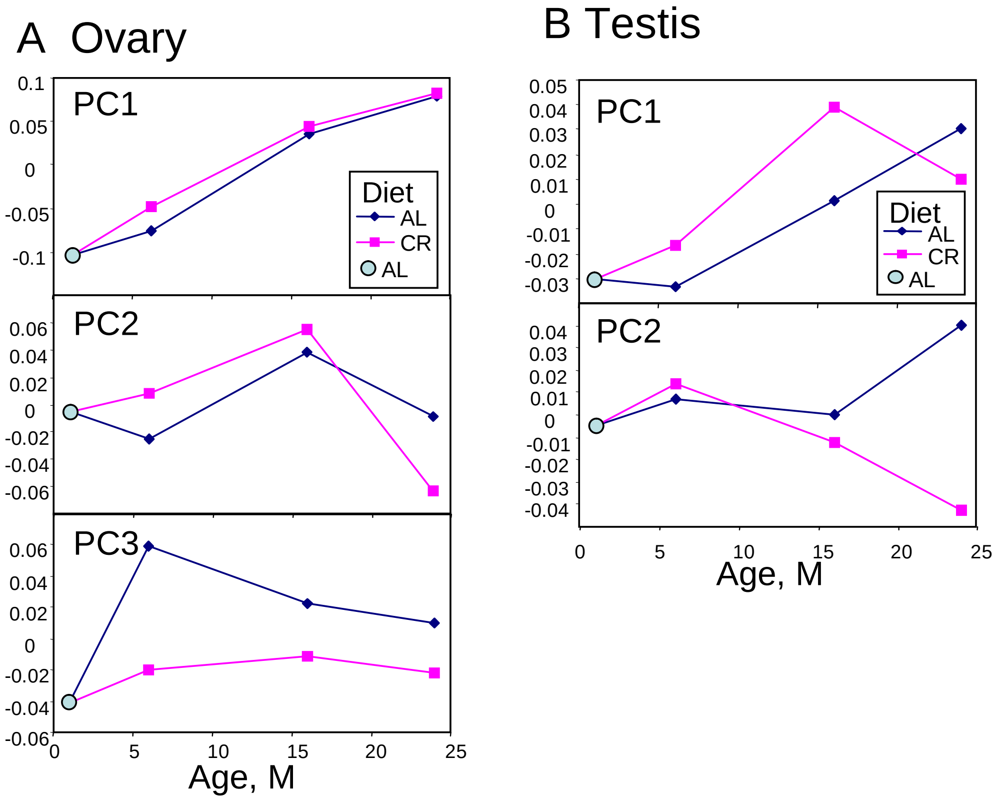

Supplemental Figure S1

Principal Component Analysis (PCA) of expression of all genes in ovary (A) and testis (B) of mice from 1 to 24 month old on *ad libitum* (AL) or calorie restriction (CR) diet. In ovary, PC1 is associated with age; PC2, is related to initial increase of gene expression followed by a decrease in the oldest age; PC3, represents the effect of calorie restriction which is strongest at the age of 6 months. In testis, PC1, is related to age; PC2, is associated with CR but mostly in the oldest animals. First 2 principal components explained 58% of variance in testis compared to 74% in ovary, which indicates that PCA patterns for testis were noisier than those for ovary due to very limited changes in gene expression values.
