# Supplementary material for: Effects of aging and calorie restriction on the global gene expression profiles of mouse testis and ovary
Source: BMC Biol. 2008 Jun 3;6:24. doi: 10.1186/1741-7007-6-24 (PMC2426674; doi:10.1186/1741-7007-6-24)
Supplement: Additional file 6 — Gene expression changes in mouse ovarywith age from 1 to 24 months on ad libitum (AL) or calorierestriction (CR) diet. Panels A-F show genes whose expression increases with age; panels G-L show genes whose expression decreases with age. Genes were selected arbitrarily to represent each functional category, but most of them were genes with the greatest differential expression in each functional category. (A) interleukin-related genes, (B) MHC-II genes, (C) complement component genes, (D) C-lectin genes, (E) apoptosis-related genes, (F) TNF receptor genes, (G) oogenesin genes, (H) zona pellucida genes, (I) other oocytes-specific genes, (J) cell cycle-related genes, (K) BMP-signaling genes, and (L) DNA repair genes. [file 1741-7007-6-24-S6.pdf]

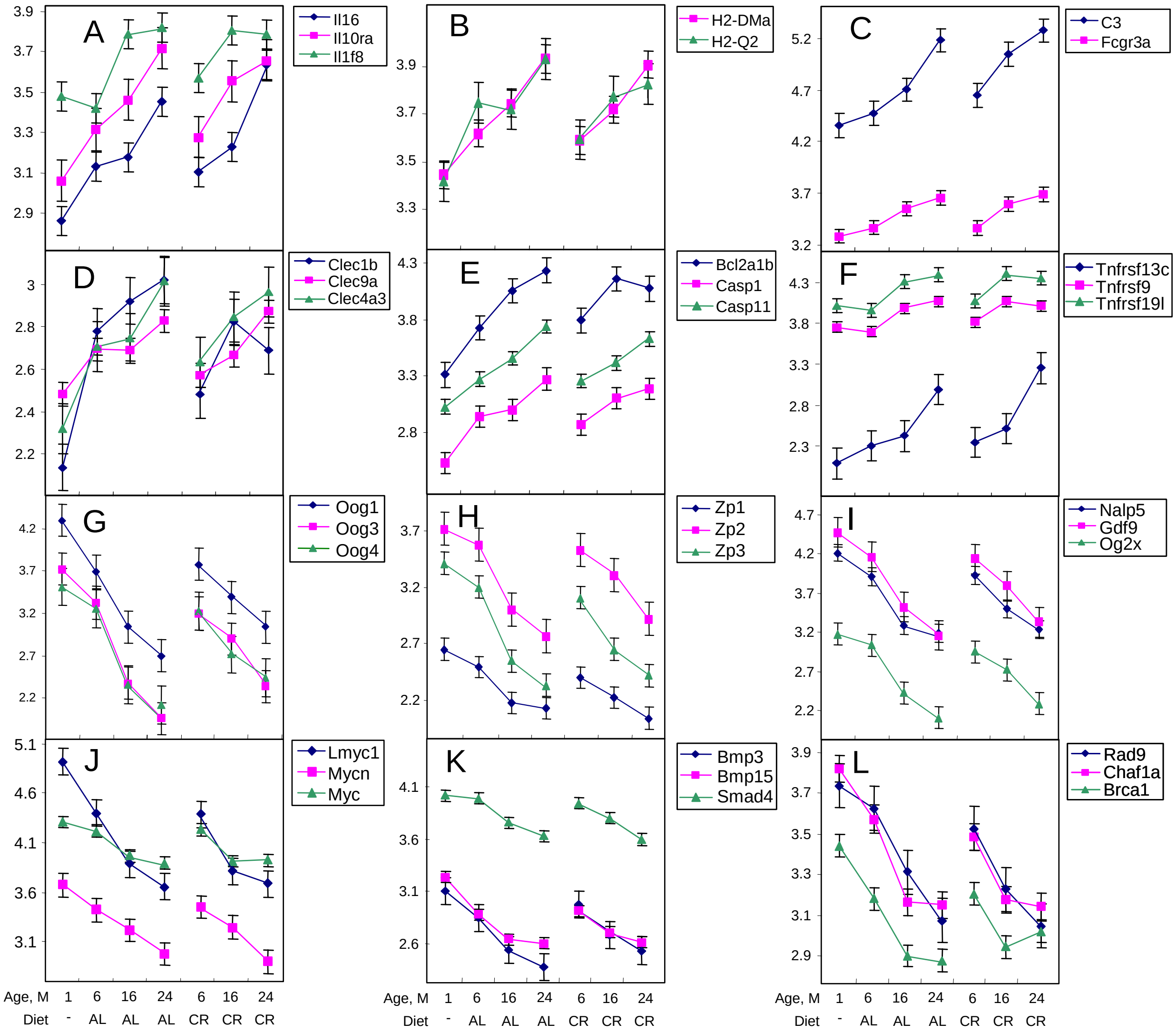

Supplemental Figure S2

Gene expression changes in mouse ovary with age from 1 to 24 month on *ad libitum* (AL) or calorie restriction (CR) diet. Panels A-F show genes whose expressions increase with age; panels G-L show genes whose expressions decrease with age. Genes were selected arbitrarily to represent each functional category, but most of them were genes with the greatest differential expressions in each functional category. (A) interleukin-related genes, (B) MHC-II genes, (C) complement component genes, (D) C-lectin genes, (E) apoptosis-related genes, (F) TNF receptor genes, (G) oogenesin genes, (H) zona pellucida genes, (I) other oocytes-specific genes, (J) cell cycle-related genes, (K) BMP-signaling genes, and (L) DNA repair genes.
