# Supplementary material for: Effects of aging and calorie restriction on the global gene expression profiles of mouse testis and ovary
Source: BMC Biol. 2008 Jun 3;6:24. doi: 10.1186/1741-7007-6-24 (PMC2426674; doi:10.1186/1741-7007-6-24)
Supplement: Additional file 8 — Gene expression changes in mouse testis with age from 1 to 24 months on ad libitum (AL) and calorie restriction (CR) diet. Panels A and B show genes whose expression increases with age; panels C and D show genes whose expression decreases with age. Genes were selected arbitrarily to represent each functional category, but most of them were genes with the greatest differential expression in each functional category. (A) immune response genes; (B) metabolism genes; and (C) extracellular matrix genes. [file 1741-7007-6-24-S8.pdf]

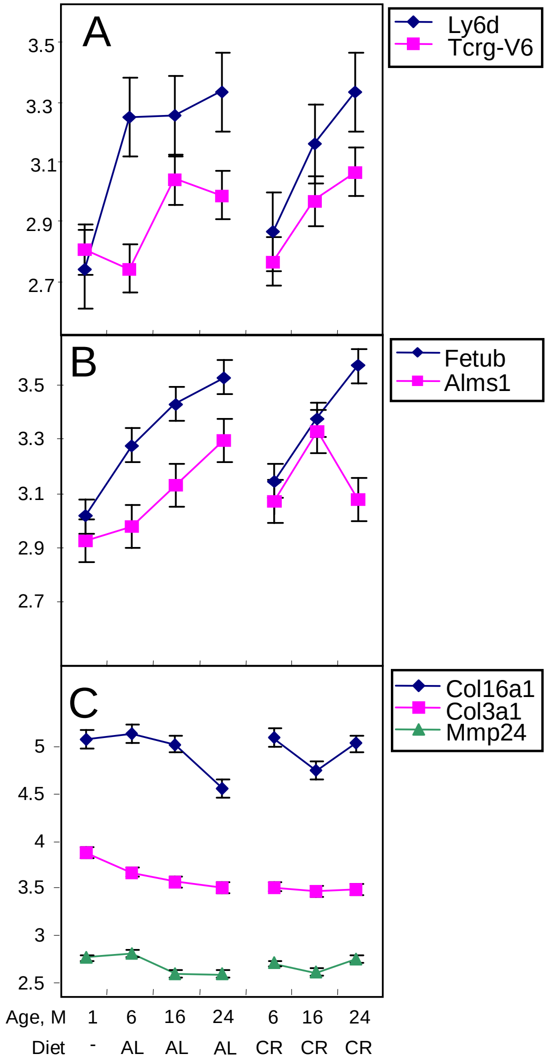

Supplemental Figure S3

Gene expression changes in mouse testis with age from 1 to 24 month on *ad libitum* (AL) and calorie restriction (CR) diet. Panels A and B show genes whose expressions increase with age; panels C and D show genes whose expressions decrease with age. Genes were selected arbitrarily to represent each functional category, but most of them were genes with the greatest differential expressions in each functional category. (A) immune response genes; (B) metabolism genes; and (C) extracellular matrix genes.
