# Supplementary material for: Effects of aging and calorie restriction on the global gene expression profiles of mouse testis and ovary
Source: BMC Biol. 2008 Jun 3;6:24. doi: 10.1186/1741-7007-6-24 (PMC2426674; doi:10.1186/1741-7007-6-24)
Supplement: Additional file 18 — Expression of genes associated with aging and DNA damage control in ovary and testis in mice from 1 to 24 months old on ad libitum (AL) or calorie restriction (CR) diet. Genes were selected arbitrarily to represent each category. (A) genes involved in human diseases, (B) sirtuin family genes, and (C) telomerase-related genes. [file 1741-7007-6-24-S18.pdf]

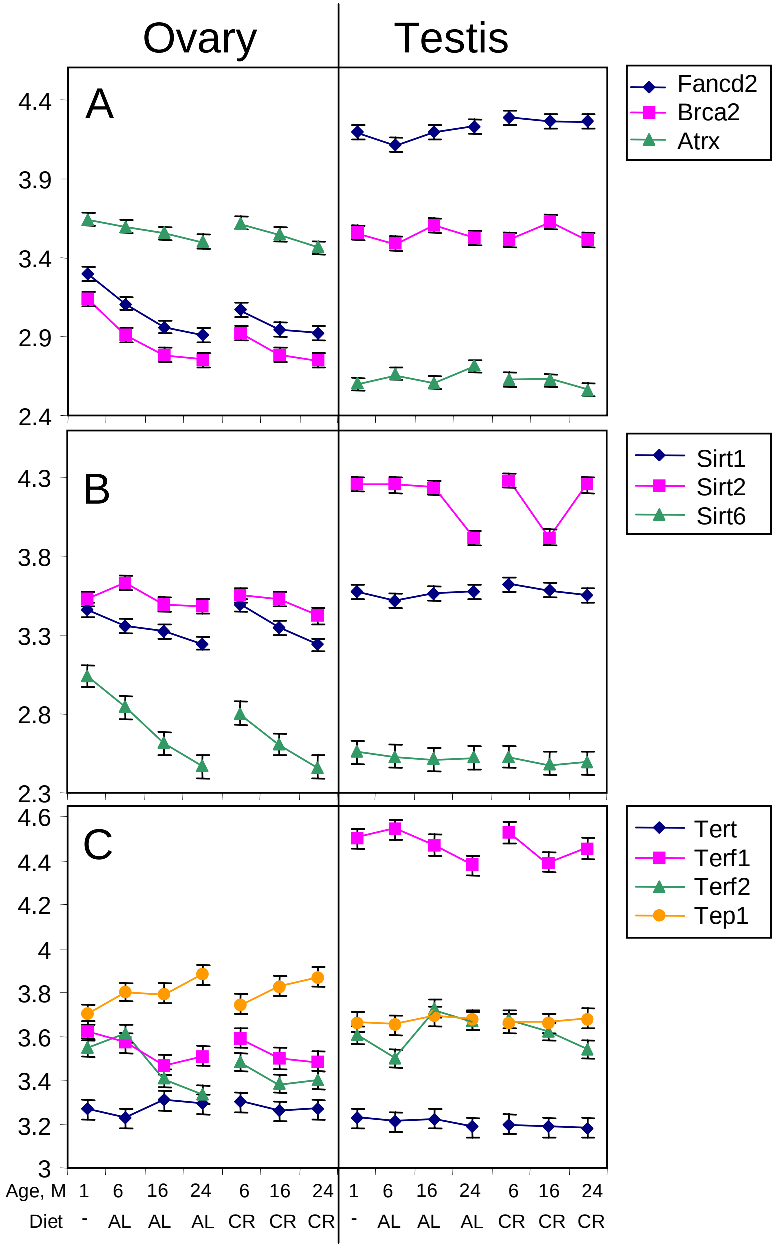

Supplemental Figure S5

Expression of genes associated with aging and DNA damage control in ovary and testis in mice from 1 to 24 month old on *ad libitum* (AL) or calorie restriction (CR) diet. Genes were selected arbitrarily to represent each category. (A) genes involved in human diseases, (B) sirtuin family genes, and (C) telomerase-related genes.
