# Supplementary figures and images for: Effects of aging and calorie restriction on the global gene expression profiles of mouse testis and ovary
Source: BMC Biol. 2008 Jun 3;6:24. doi: 10.1186/1741-7007-6-24 (PMC2426674; doi:10.1186/1741-7007-6-24)

# Ovary

# Ovary

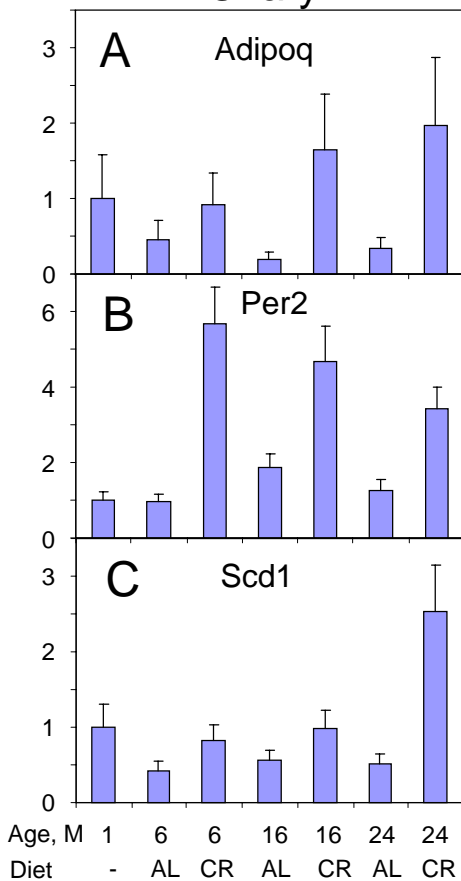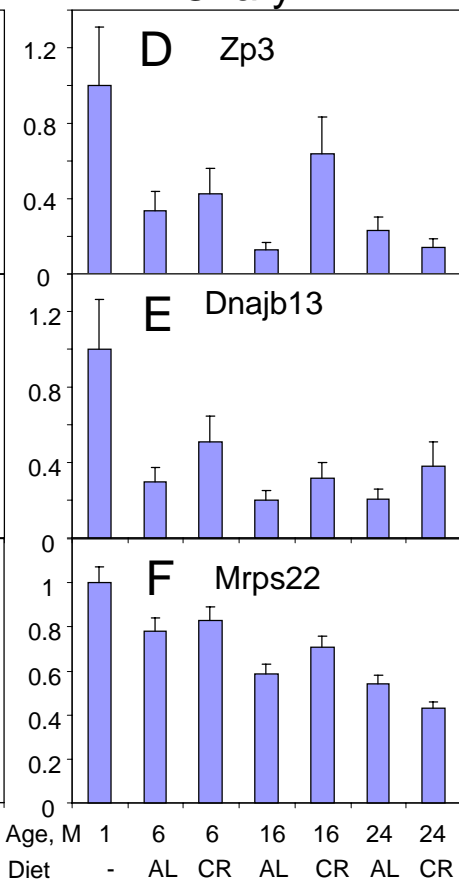

# Testis

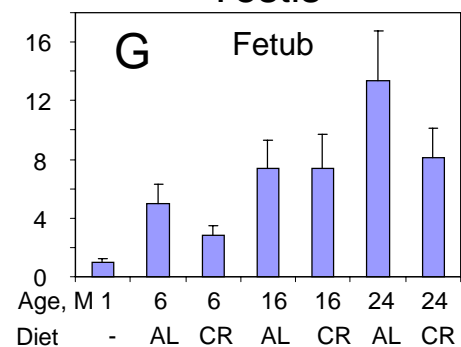

Supplement: Additional file 20 — Quantitative RT-PCR analysis of selected genes in ovary and testis in mice from 1 month to 24 months on AL or CR diet. [file 1741-7007-6-24-S20.pdf]
